# NLRP3/Caspase-1 Pathway-Induced Pyroptosis Mediated Cognitive Deficits in a Mouse Model of Sepsis-Associated Encephalopathy

**DOI:** 10.1007/s10753-018-0894-4

**Published:** 2018-10-01

**Authors:** Qun Fu, Jing Wu, Xiao-Yan Zhou, Mu-Huo Ji, Qing-Hong Mao, Qing Li, Man-Man Zong, Zhi-Qiang Zhou, Jian-Jun Yang

**Affiliations:** 1 Department of Anesthesiology, Jinling Hospital, Medical College of Nanjing Medical University, Nanjing, 210002 China; 2 Department of Anesthesiology, Affiliated Hospital of Integrated Traditional Chinese and Western Medicine, Nanjing University of Chinese Medicine, Nanjing, 210028 China; 3 Jiangsu Key Laboratory of Molecular Medicine, Medical School of Nanjing University, Nanjing, 210093 China; 4 Department of Anesthesiology, The first Affiliated Hospital of Zhengzhou University, Zhengzhou, 450000 China; 5 Department of Anesthesiology, Zhongda Hospital, Medical School, Southeast University, Nanjing, 210009 China

**Keywords:** NLRP3, caspase-1, pyroptosis, pro-inflammatory cytokine, cognitive impairment

## Abstract

Sepsis-associated encephalopathy (SAE) is a common complication that leads to long-term cognitive impairments and increased mortality in sepsis survivors. The mechanisms underlying this complication remain unclear and an effective intervention is lacking. Accumulating evidence suggests the nucleotide-binding domain-like receptor protein3 (NLRP3)/caspase-1 pathway is involved in several neurodegenerative diseases. Thus, we hypothesized that the NLRP3/caspase-1 pathway is involved in NLRP3-mediated pyroptosis, maturation and release of inflammatory cytokines, and cognitive deficits in SAE. We used the NLRP3 inhibitor MCC950 and the caspase-1 inhibitor Ac-YVAD-CMK to study the role of the NLRP3/caspase-1 pathway in pyroptosis and cognitive deficits in a mouse model of SAE. Mice were randomly assigned to one of six groups: sham+saline, sham+MCC950, sham+Ac-YVAD-CMK, cecal ligation and puncture (CLP)+saline, CLP+MCC950, and CLP+Ac-YVAD-CMK. Surviving mice underwent behavioral tests or had hippocampal tissues collected for histochemical analysis and biochemical assays. Our results show that CLP-induced hippocampus-dependent memory deficits are accompanied by increased NLRP3 and caspase-1 positive cells, and augmented protein levels of NLRP3, caspase-1, gasdermin-D, and pro-inflammatory cytokines in the hippocampus. In addition, administration of MCC950 or Ac-YVAD-CMK rescues cognitive deficits and ameliorates increased hippocampal NLRP3-mediated neuronal pyroptosis and pro-inflammatory cytokines. Our results suggest that the NLRP3/caspase-1 pathway-induced pyroptosis mediates cognitive deficits in a mouse model of SAE.

## INTRODUCTION

Sepsis-associated encephalopathy (SAE), which is characterized by long-term cognitive impairments and psychiatric diseases in sepsis survivors, is associated with increased morbidity and mortality [1–4]. Several mechanisms are involved in the pathogenesis of SAE, such as endothelial activation, disturbance of the blood-brain barrier, oxidative damage, neurotransmission disturbances, altered brain signaling, and neuronal apoptosis [5]. There is accumulating evidence that neuroinflammation plays a key role in the development of SAE [1, 6]. Nonetheless, the mechanisms by which sepsis induces overactivated neuroinflammation have yet to be elucidated.

Pyroptosis is an inflammatory form of programmed cell death and plays a protective role in host defense during infection. This kind of cell death is characterized by cell swelling, lysis, and release of cytoplasmic content. The initiation of pyroptosis leads to pore-mediated cell lysis and IL-1β secretion [7]. However, excessive pyroptosis is harmful to normal tissues and cells. To avoid host organism damage, pyroptosis is tightly regulated by the activation of inflammatory caspases, such as caspase-1, caspase-4, caspase-5, and caspase-11. These caspases found in the canonical and non-canonical inflammasome signaling pathways lead to inflammatory responses [8–10]. However, it remains unclear how these caspases initiate pyroptosis.

The canonical inflammasome pathway is triggered by various cytoplasmic sensor proteins that recognize inflammatory agents and multiple pathogens and recruit pro-caspase-1 monomers by the apoptosis-associated speck-like protein containing a CARD (ASC) and activate caspase-1 through dimerization. The non-canonical inflammasome pathway is activated by lipopolysaccharide molecules in the cytoplasm of infected cells [11]. The nucleotide-binding domain-like receptor protein 3 (NLRP3) inflammasome is an intracellular protein complex that plays a crucial role in innate immune sensing. Moreover, activation of NLRP3 leads to the maturation of caspase-1, which mediates pyroptosis [12] and regulates the cleavage and maturation of pro-inflammatory cytokines, such as IL-1β and IL-18 [13]. However, how much pyroptosis and increased inflammatory cytokines are involved in the cognitive impairments that occur during SAE remains unknown.

In light of these findings, we hypothesize that sepsis triggers NLRP3-mediated pyroptosis, neuro-inflammatory responses, and cognitive deficits through the NLRP3/caspase-1 pathway, and that administration of the NLRP3 inhibitor MCC950 and the caspase-1 inhibitor Ac-YVAD-CMK could attenuate hippocampal neuronal pyroptosis and neuroinflammation, reducing long-term cognitive deficits and improving survival rates.

## MATERIALS AND METHODS

### Animals

One hundred and seventy-four C57BL/6 male mice aged 4 months were purchased from the Animal Center of Jinling Clinical Medical College at Nanjing Medical University, Nanjing, China. All experimental procedures in this study were performed according to the Guidelines for the Care and Use of Laboratory Animals from the National Institutes of Health, USA. Experiments began after mice had acclimated to the environment for 2 weeks. Mice were housed in groups of five individuals per cage with a 12:12 h light:dark cycle at a temperature of 23–25 °C with food and water available *ad libitum*.

### Surgical Procedures

Mice were subjected to cecal ligation and puncture (CLP) as previously described [14]. Briefly, mice were intraperitoneally anesthetized using 2% sodium pentobarbital (40 mg/kg; Sigma Chemical Co, St. Louis, MO, USA). Under aseptic conditions, the cecum was carefully isolated and then ligated with 4.0 silk below the ileocecal junction, approximately 1.2 cm from the distal end. The cecum was then perforated twice with a sterile 22-gauge needle and gently squeezed to extrude fecal contents into the peritoneal cavity. After that, the cecum was returned to the peritoneal cavity and the laparotomy incision closed with 4.0 silk sutures. Mice subjected to sham operation had the cecum exposed in the same way as for CLP but it was neither ligated nor punctured. Mice were revived immediately after surgery by administering regular saline (30 ml/kg) subcutaneously and returned to their cages.

### Drug Administrations

Mice were randomly divided into one of the following six groups: sham+saline (*n* = 18), sham+MCC950 (*n* = 18), sham+Ac-YVAD-CMK (*n* = 18), CLP+saline (*n* = 40), CLP+MCC950 (*n* = 40), and CLP+Ac-YVAD-CMK (*n* = 40). Saline, MCC950 (10 mg/kg, China Peptides Co. Ltd., China) [15], or Ac-YVAD-CMK (100 μg per mouse, Cayman Chemical Company, USA) [16] was intraperitoneally injected 30 min before surgery and on days 1, 2, 4, and 6 after surgery. Seven days after surgery, six mice in each group were deeply anesthetized using 2% sodium pentobarbital (60 mg/kg) and decapitated. The brain was rapidly removed and separated into two halves for histochemical analysis and biochemical assays. The remaining mice in each group were used in behavioral tests 2 weeks after surgery.

### Hematoxylin and Eosin (HE) Staining

Half of each mouse brain harvested (*n* = 6 for each group) underwent HE staining and immunohistochemical analysis. Brain tissues were immersed in 4% paraformaldehyde and embedded using paraffin. The tissues were sliced into 4-mm sections until use.

HE staining was performed as follows: hematoxylin staining for 5 min; 75% hydrochloric acid alcohol solution for 30 s decoloring; eosin staining for 5 min; and, 90% ethanol for 35 s decoloring. Normal neurons have a relatively large cell body that is rich in cytoplasm with one or two big round nuclei, while damaged cells show shrunken cell bodies, pyknotic nuclei, dark cytoplasm, and many empty vesicles. Hippocampal neuronal damage was evaluated using a standard semi-quantitative scale [17]. In brief, grade 0, no damage to any hippocampal subregion; grade 1, scattered neurons are damaged in the CA1 subregion; grade 2, moderate numbers of damaged neurons in the CA1 subregion (< 50% neurons damaged); grade 3, severe damage (> 50% of cells affected) to pyramidal cells in the CA1 subregion; and grade 4, extensive cell damage in all hippocampal regions. Four random high-power (× 400) visual fields from each brain slice were checked. Evaluation of cells with nuclear pyknosis and morphologic abnormality was performed by two pathologists blind to the treatment groups.

### Immunohistochemistry

Paraffin sections were then deparaffinized and hydrated using the following steps: 10 min in xylene twice; 5, 10, 10, and 10 min in 100%, 95%, 85%, and 70% ethanol, respectively; and 5 min in PBS at room temperature repeated three times. Antigen retrieval was achieved by boiling the sections in 10 mM sodium citrate for 10 min in a microwave oven. Sections were then washed with PBS three times and treated with 3% H_2_O_2_-methanol for 15 min. Immunostaining was performed by incubation with antibodies against NLRP3 (1:100; Servicebio Technology Co. Ltd., Wuhan, China) and caspase-1 (1:100; Servicebio Technology Co. Ltd., Wuhan, China); cells with brownish yellow cytoplasm were recorded as positive cells. The numbers of NLRP3 and caspase-1 immunoreactive cells in the hippocampal CA1 region were measured by an investigator blind to group assignment.

### Western Blotting Analysis

The hippocampus was harvested from the second brain half from each animal (*n* = 6 for each group) and homogenized for biochemical assays. Proteins from each hippocampus were electrophoretically separated and blotted onto nitrocellulose membranes. Protein levels were estimated *via* incubation against antibodies of NLRP3 (1:600; Servicebio Technology Co. Ltd.), ASC (1:200; Santa Cruz, USA), cleaved caspase-1 (1:500; Servicebio Technology Co. Ltd.), gasdermin-D (GSDMD, 1:500; Biorbyt, UK), IL-1β (1:200; Santa Cruz, USA), IL-18 (1:200; Santa Cruz, USA), and β-actin (1:5000; Bioworld, USA). Protein bands were visualized *via* enhanced chemiluminescence and quantified using ImageQuant software (Syngene).

### Enzyme-Linked Immunosorbent Assay

Hippocampal IL-1β and IL-18 levels were quantified using an enzyme-linked immunosorbent assay (ELISA) kit according to the manufacturer’s directions (Servicebio Technology Co. Ltd).

### OpenField Test

Behavioral tests were conducted on surviving mice 2 weeks after surgery in a sound-isolated room by a single investigator that was blind to treatment group assignment. All tests were conducted between 14:00 and 17:00 h. Locomotor and exploratory activities of mice were measured in an open-field apparatus [18]. A mouse was gently placed in the center of a white plastic chamber (50 × 50 × 30 cm) for 5 min and activities were automatically recorded using a video tracking system (XR-XZ301, Shanghai Xinruan Soft Information Technology Co. Ltd., Shanghai, China). The apparatus was cleaned with 75% alcohol between tests to remove any odor cues.

### Fear Conditioning Test

Two hours after the open-field test, mice were trained for a fear conditioning test, as described previously [18]. Each mouse was gently placed into a conditioning chamber (XRXC404, Shanghai Soft Maze Information Technology Co., Ltd., Shanghai, China) and allowed to acclimate for 3 min. A 30-s tone (75 dB, 3 kHz) was then delivered followed by a 2-s foot shock (0.75 mA). The mouse was kept in the chamber for another 30 s and then returned to its home cage. A context test to evaluate hippocampus-dependent memory was performed 24 h after training. Each mouse was returned to the same test chamber for 5 min without any stimulation. After the context test, each mouse was placed for 390 s in a novel chamber altered in shape, color, and smell. The same tone was presented for another 3 min without the foot shock to evaluate hippocampus-independent memory. Cognitive deficits in the test was assessed by measuring the amount of time the mouse demonstrated “freezing behavior,” which is defined as a completely immobile posture except for respiratory efforts. Freezing behavior was automatically recorded by a video tracking system.

### Statistical Analysis

Statistical analyses were done using the software Statistical Product for Social Sciences (SPSS; version 16.0, Chicago, IL, USA) and data plotted using GraphPad Prism 5.0 Software (San Diego, CA, USA). Survival rate was assessed by the Kaplan-Meier method and compared among treatment groups using the log-rank test. Differences among groups were tested using a one-way analysis of variance followed by Tukey’s tests. Data are expressed as mean ± SEM. A *p* value < 0.05 was considered statistically significant.

## RESULTS

### MCC950 or Ac-YVAD-CMK Improved Survival Rate of CLP Mice

**Fig. 1:**
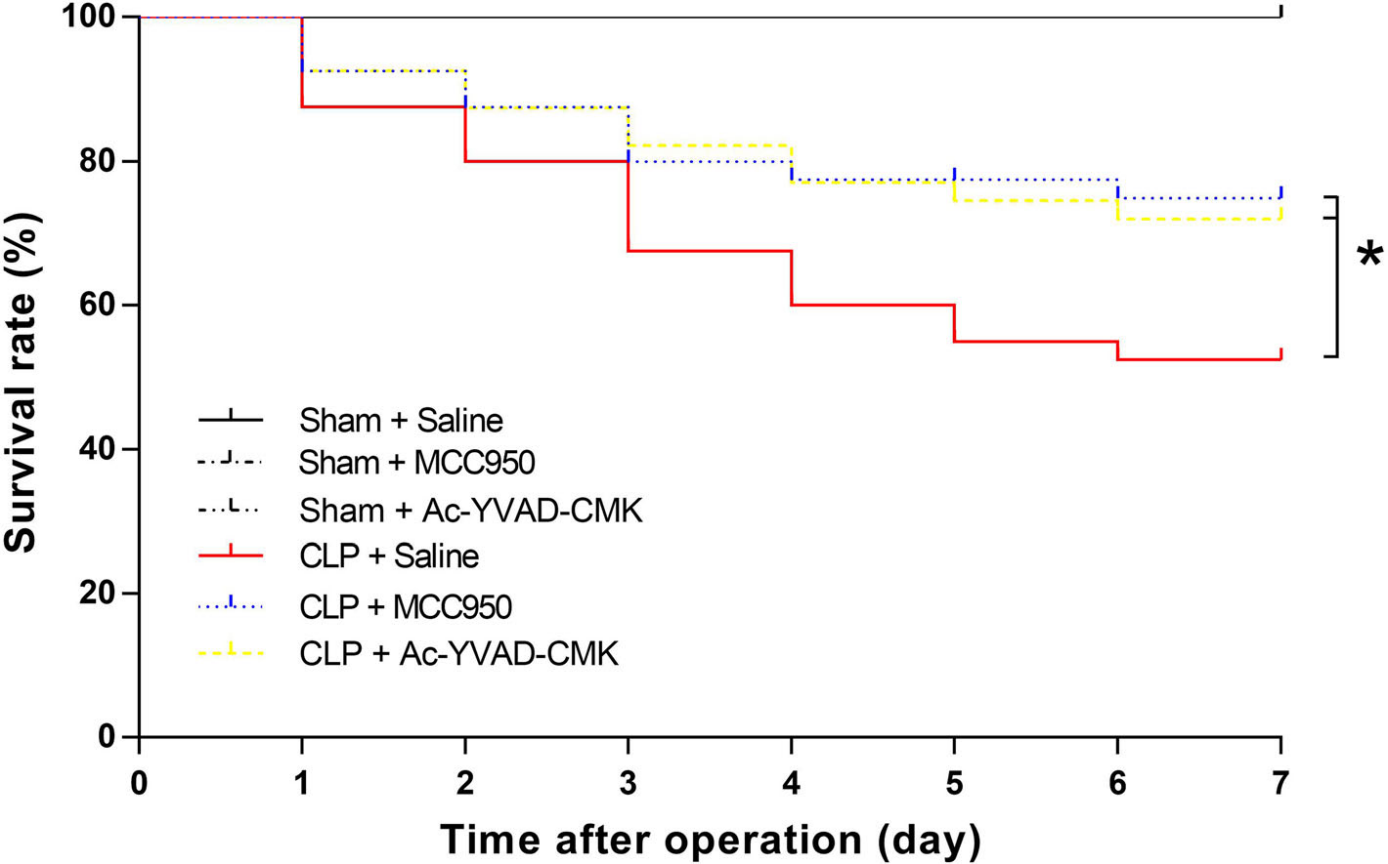
MCC950 or Ac-YVAD-CMK improved survival rate in the first 7 days after CLP. Mice were randomly divided into six groups: sham+saline, sham+MCC950, sham+Ac-YVAD-CMK, CLP+saline, CLP+MCC950, and CLP+Ac-YVAD-CMK. Normal saline (0.25 ml per mouse), MCC950 (10 mg/kg), or Ac-YVAD-CMK (100 μg per mouse) was administered to mice intraperitoneally 30 min before surgery and on days 1, 2, 4, and 6 after surgery. Survival rate to day 7 was evaluated (*n* = 18–40 mice/group). The asterisk indicates *P* < 0.05 *versus* the CLP+saline group.

Mouse mortality increased for 7 days after CLP, confirming the results of our previous study [14]. In this study, survival rate increased with administration of intraperitoneal MCC950 (77.5%) or Ac-YVAD-CMK (75%), compared to the CLP + saline group (52.5%; Fig. 1). This indicates that MCC950 and Ac-YVAD-CMK improve the survival rate of CLP mice.

### MCC950 or Ac-YVAD-CMK Attenuated Hippocampus-Dependent Memory Impairments in SAE Mice

**Fig. 2:**
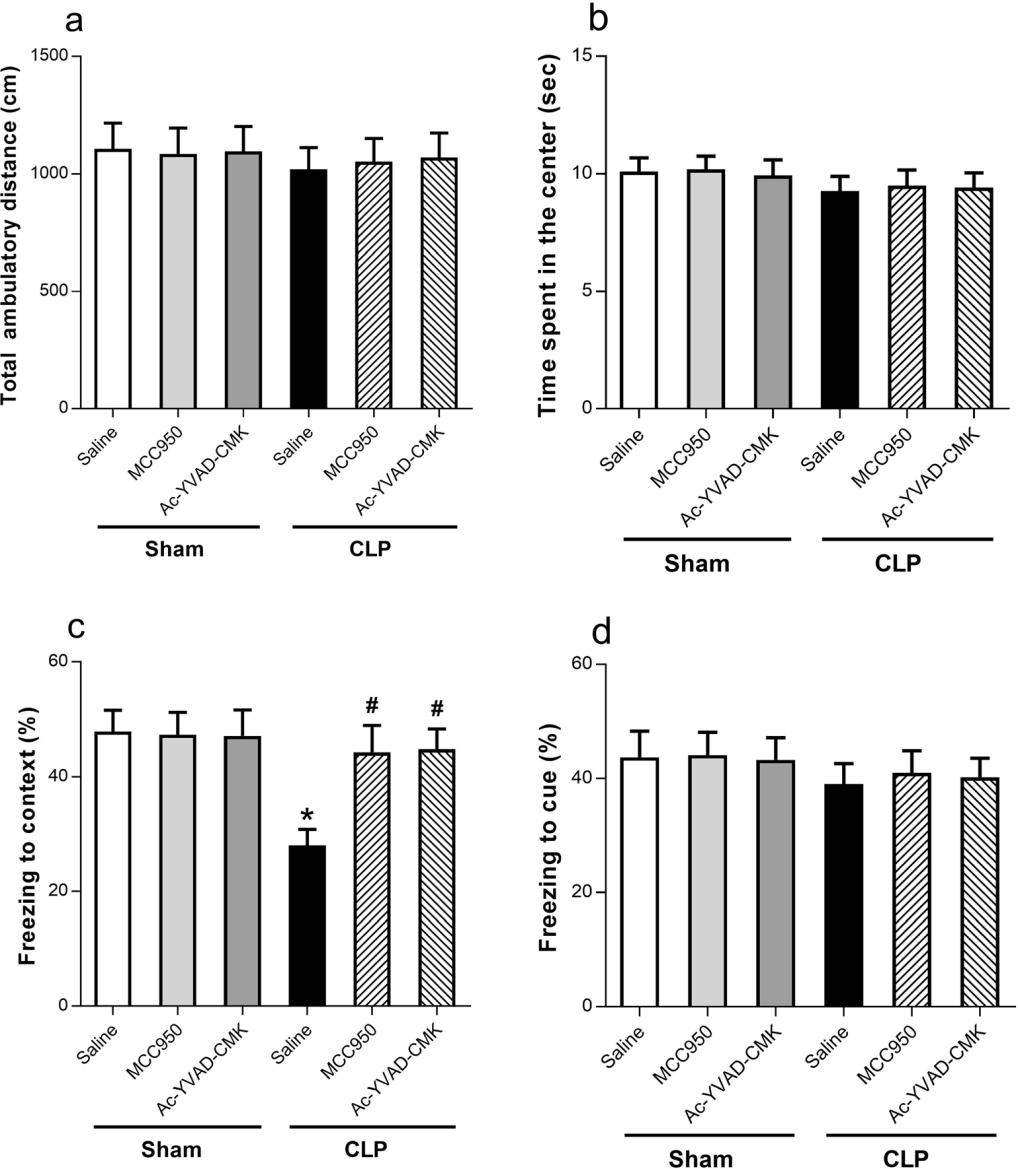
MCC950 or Ac-YVAD-CMK reversed CLP-induced learning and memory impairments. **a** Total ambulatory distance. **b** Time spent in the center. **c** The contextual fear conditioning test. **d** The cued fear conditioning test. Data are presented as the mean ± SEM (*n* = 12–24 mice/group). The asterisk indicates *P <* 0.05 *versus* the sham+saline group; the pound sign, *P <* 0.05 *versus* the CLP+saline group. For the procedures of the open-field test and fear conditioning test, see “MATERIALS AND METHODS.”

Mice recover with no signs of infection or motor alterations by 10 days post-CLP [19]. Thus, behavior tests were done 2 weeks after surgery. The total distance moved and time spent in the center of the arena were similar among all six groups (Fig. 2a, b).

A fear conditioning test was used to evaluate whether CLP-induced long-term memory impairments were present 24 h after training. The CLP+saline group had a shorter freezing time of context test than the sham+saline group, indicating the presence of a sepsis-induced hippocampus-dependent long-term memory deficit. However, this memory deficit was ameliorated by administration of MCC950 or Ac-YVAD-CMK (Fig. 2c). No differences were observed in freezing time of the cue test among the six groups (Fig. 2d), indicating that hippocampus-independent memory deficit was not found in the SAE mice. Our results suggest that MCC950 and Ac-YVAD-CMK have a therapeutic effect on CLP-induced cognitive deficits.

### MCC950 or Ac-YVAD-CMK Rescued Morphological Damages of Hippocampus in SAE Mice

**Fig. 3:**
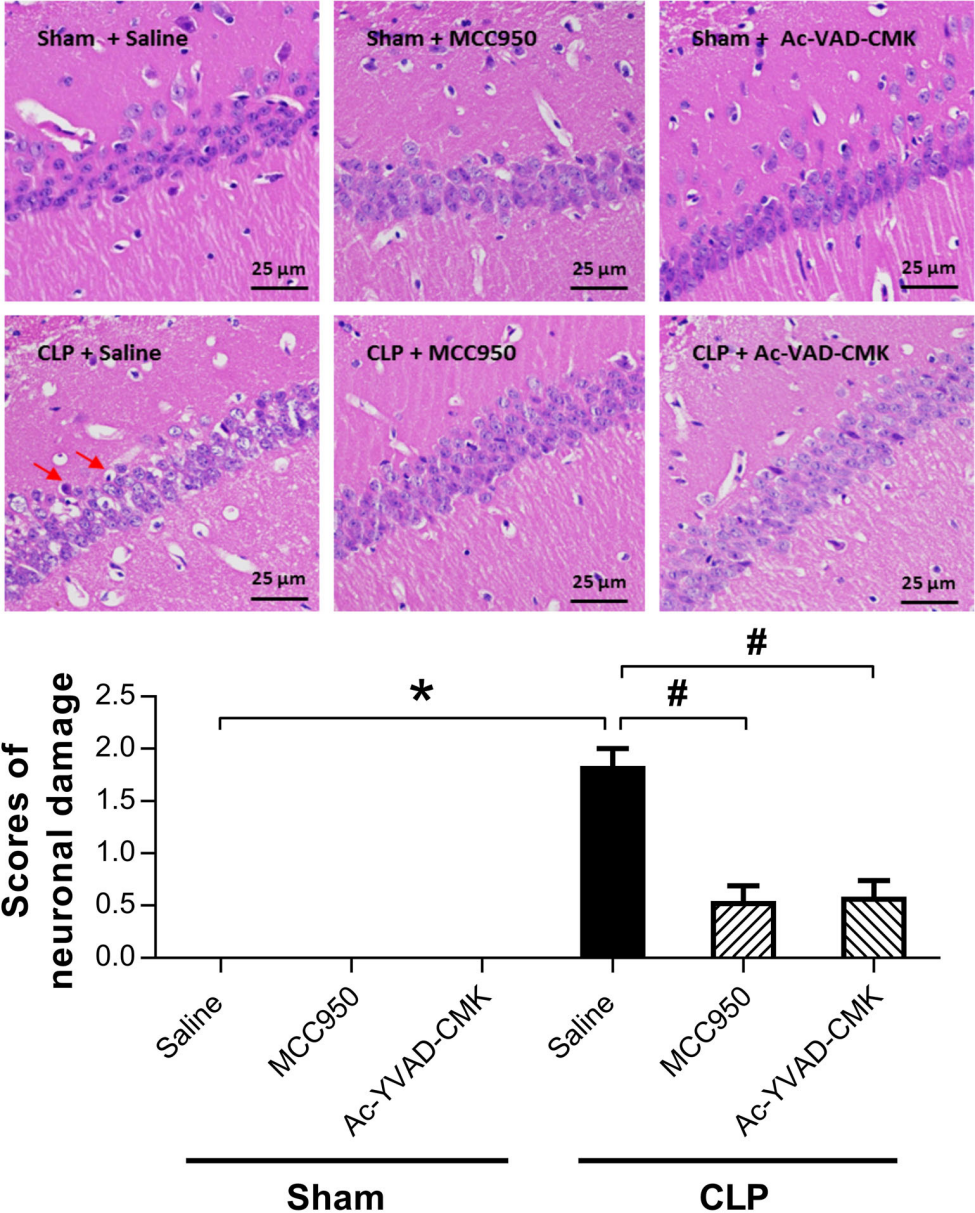
MCC950 or Ac-YVAD-CMK reduced CA1 neuronal damages in the hippocampus of SAE mice. **a** Representative pictures of HE staining showing morphologic abnormality of pyramidal cells in the hippocampus CA1 region. Pyramidal cells had an orderly arrangement and had complete cell structure in the sham groups; in CLP+saline group, cells had a disorderly arrangement, with the cytoplasm and nucleolus dyed deeply indicating nuclear pyknosis (indicated by arrows). The number of degenerate cells in the CLP+MCC950 group and CLP+Ac-YVAD-CMK groups was reduced relative to the CLP+saline group. Scale bar indicates 25 μm. **b** Semi-quantitative analysis shows significant reduction in hippocampal neuronal damage for mice treated with MCC950 or Ac-YVAD-CMK compared to mice treated with saline after CLP. Data are presented as the mean ± SEM (*n* = 6 mice/group). The asterisk indicates *P <* 0.05 *versus* the sham+saline group; the pound sign, *P <* 0.05 *versus* the CLP+saline group.

To evaluate sepsis-induced histological damage and the neuroprotective effects of MCC950 and Ac-YVAD-CMK, we did HE staining on brain sections of the hippocampus CA1 region 7 days after surgery. No abnormalities in pyramidal cell morphology were observed in the sham-operated groups. The CLP+saline group showed the most shrunken cell bodies and largest amount of nuclear pyknosis (Fig. 3). Moreover, mice given MCC950 or Ac-YVAD-CMK showed significantly fewer abnormal neurons and lower scores of neuronal damage compared to the CLP+saline group (Fig. 3). This indicates that MCC950 or Ac-YVAD-CMK can rescue hippocampal neurons from CLP-induced morphological damage.

### MCC950 or Ac-YVAD-CMK Inhibited the Activation of NLRP3/Caspase-1 Pathway in the Hippocampus of SAE Mice

**Fig. 4:**
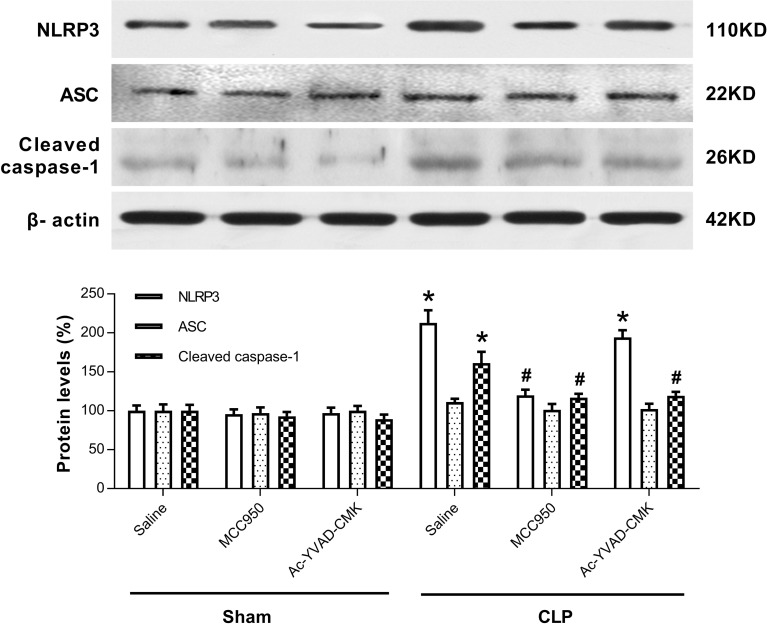
MCC950 or Ac-YVAD-CMK inhibited the activation of NLRP3/caspase-1 pathway in the hippocampus of SAE mice. Representative Western blot and quantitative analysis of protein levels of NLRP3, ASC, and caspase-1 in hippocampal tissues. Data are shown as mean ± SEM (*n* = 6 mice/group). The asterisk indicates *P <* 0.05 *versus* the sham+saline group; the pound sign, *P <* 0.05 *versus* the CLP+saline group.

**Fig. 5:**
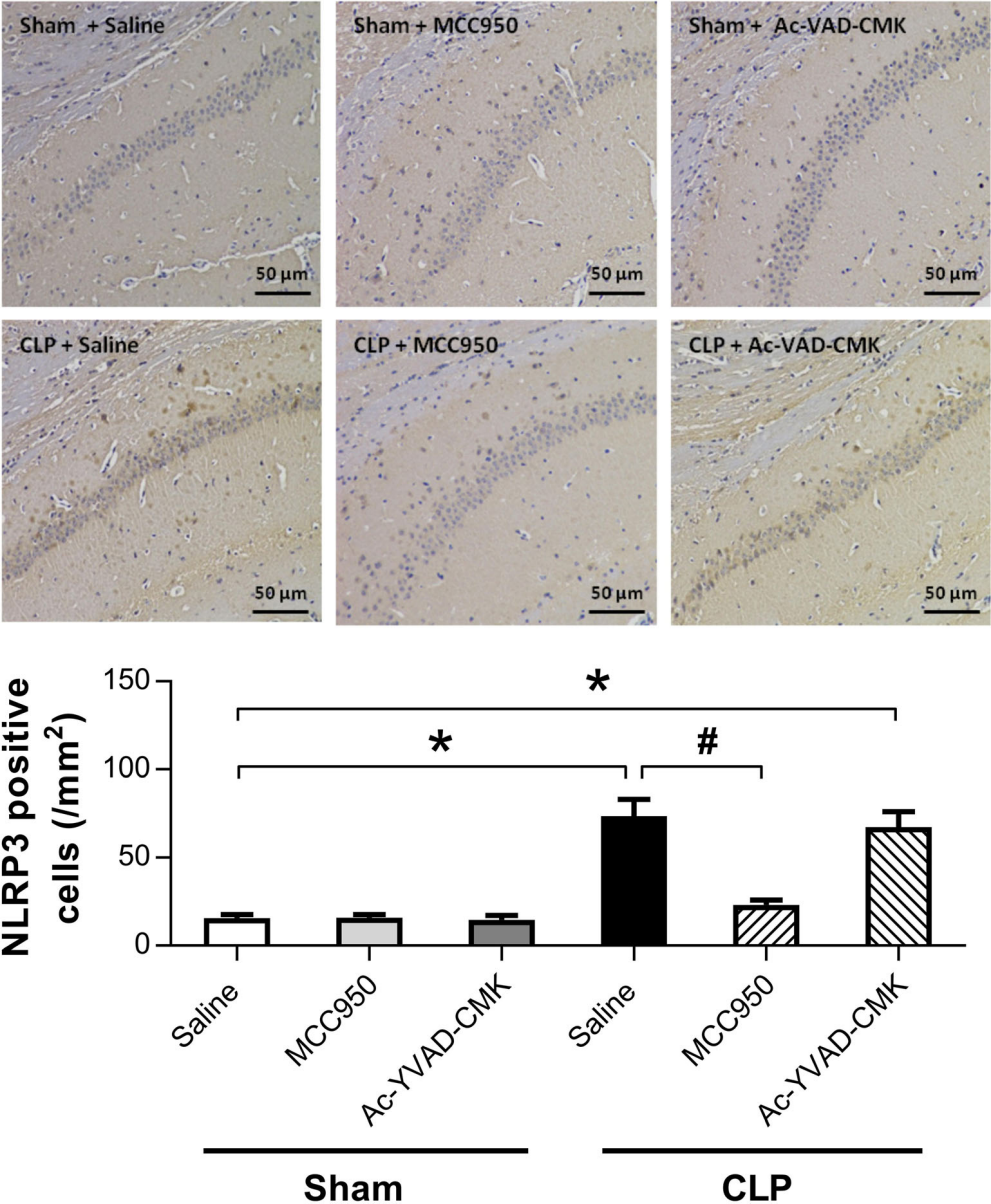
Effects of MCC950 and Ac-YVAD-CMK on the number of NLRP3 positive cells in the hippocampal CA1 region. Representative images of NLRP3 immunohistochemical (IHC) staining in the hippocampal CA1 region. Cells with brownish-yellow cytoplasm are positive for NLRP3. Scale bar indicates 50 μm. Lower panel presents statistics for the six experimental groups. Data are shown as mean ± SEM (*n* = 6 mice/group). The asterisk indicates *P <* 0.05 *versus* the sham+saline group; the pound sign, *P <* 0.05 *versus* the CLP+saline group.

**Fig. 6:**
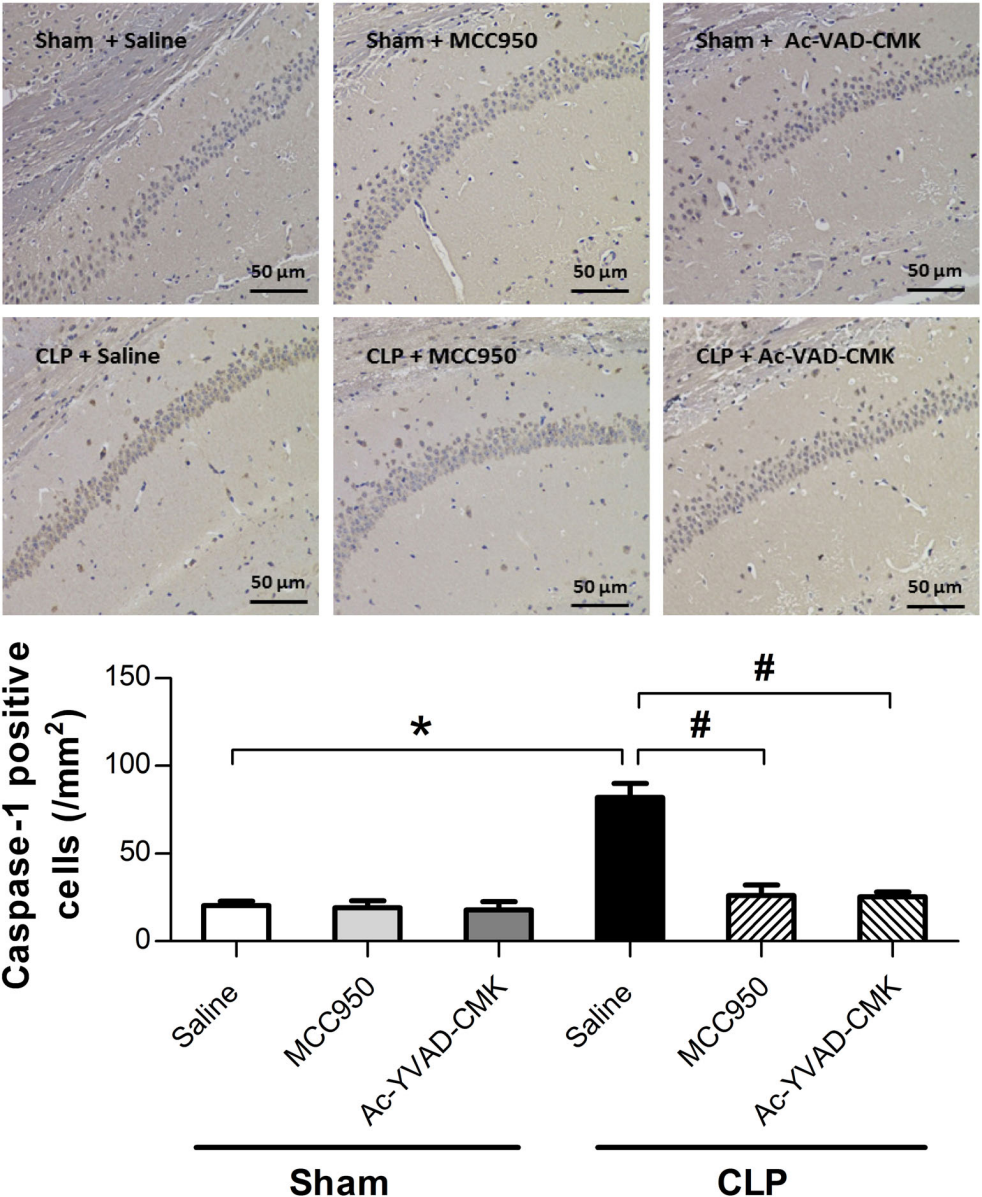
Effects of MCC950 and Ac-YVAD-CMK on the number of caspase-1 positive cells in the hippocampal CA1 region. Representative images of caspase-1 immunohistochemical (IHC) staining in the hippocampal CA1 region. Cells with brownish-yellow cytoplasm are positive for caspase-1. Scale bar indicates 50 μm. Lower panel presents statistics for the six experimental groups. Data are shown as mean ± SEM (*n* = 6 mice/group). The asterisk indicates *P <* 0.05 *versus* the sham+saline group; the pound sign, *P <* 0.05 *versus* the CLP+saline group.

The NLRP3 inflammasome, a component of the inflammatory process, is highly expressed in various neurodegenerative disorders [20–22]. CLP induces the activation of NLRP3 inflammasome in the hippocampus of SAE mice [14]. To explore whether MCC950 or Ac-YVAD-CMK inhibits the activation of the NLRP3/caspase-1 pathway, we measured levels of two markers of the inflammasome, NLRP3 and ASC, that may further activate caspase-1 [12, 23]. Our results showed that the protein levels of NLRP3 and cleaved caspase-1 were higher at 7 days post-CLP, and that this increase did not occur when MCC950 was administered (Fig. 4). Ac-YVAD-CMK treatment rescued the increase of cleaved caspase-1 but not that of NLRP3 (Fig. 4). No difference was observed in the levels of ASC among the six groups (Fig. 4). Immunohistochemical analysis revealed a significant increase in the number of cells positive for NLRP3 and caspase-1 in the CA1 region of the mouse brain at 7 days post-CLP, and that this was reversed by administration of MCC950 (Fig. 5). Ac-YVAD-CMK treatment rescued the increase of caspase-1, but not that of NLRP3 (Fig. 6). These results suggest the CLP-induced activation of NLRP3/caspase-1 pathway is inhibited by the specific inhibitors MCC950 and Ac-YVAD-CMK.

### MCC950 or Ac-YVAD-CMK Attenuated NLRP3/Caspase-1-Mediated Pyroptosis and Inflammatory Cytokines in SAE Mice

**Fig. 7:**
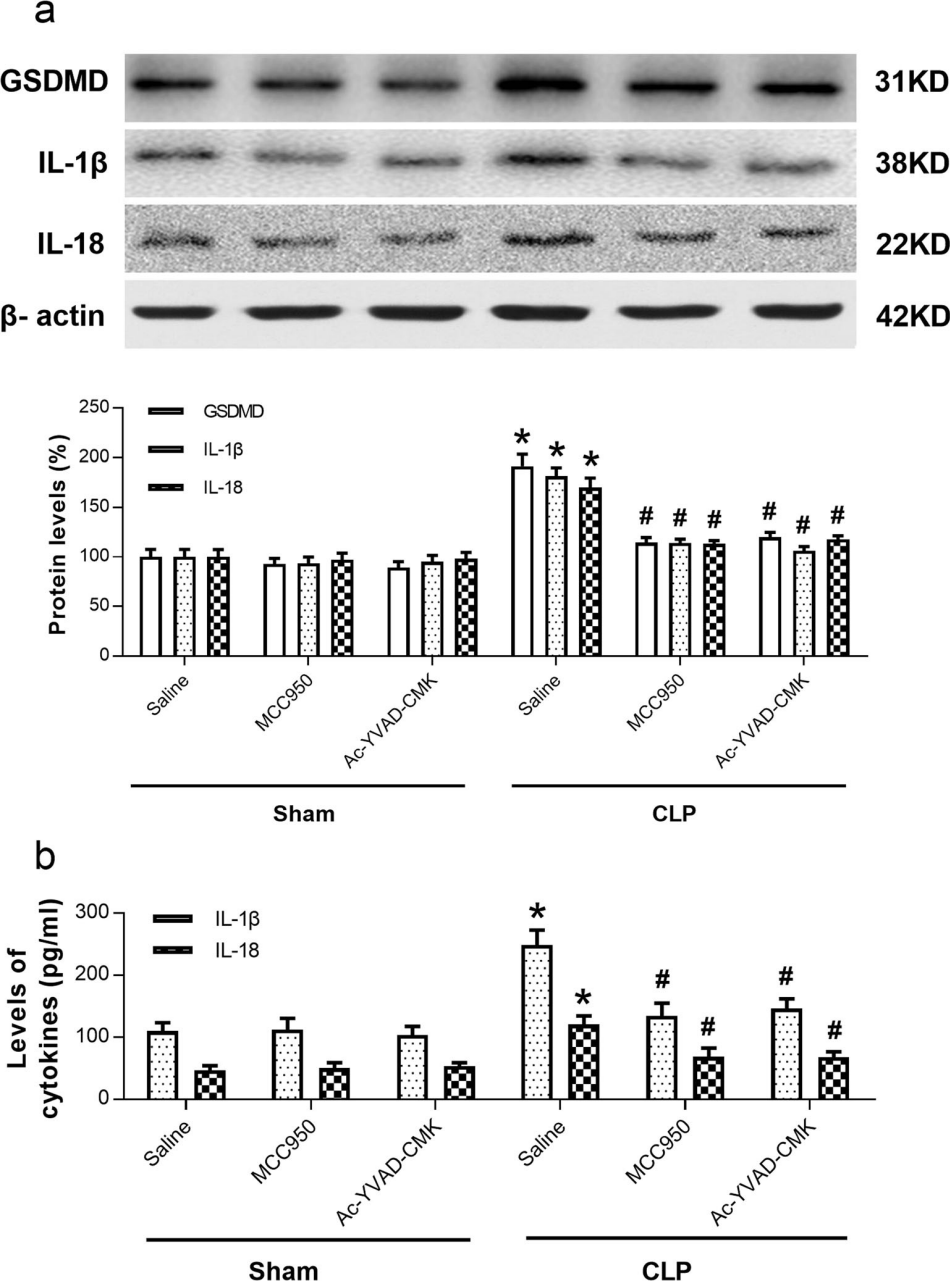
MCC950 or Ac-YVAD-CMK inhibited NLRP3/caspase-1-dependent pyroptosis and inflammatory cytokines in the hippocampus of SAE mice. **a** Representative Western blot and quantitative analysis of protein levels of GSDMD, IL-1β, and IL-18 in hippocampal tissues. **b** ELISA assays of IL-1β and IL-18 levels. Data are shown as mean ± SEM (*n* = 6 mice/group). The asterisk indicates *P <* 0.05 *versus* the sham+saline group; the pound sign, *P <* 0.05 *versus* the CLP+saline group.

The progressive dysfunction and death of neurons provide a molecular and cellular basis for memory deficits in SAE. Thus, we further investigated the role of NLRP3/caspase-1-mediated pyroptosis in cognitive deficit of SAE mice [24–26]. GSDMD is identified as the executioner of pyroptosis [27–29]. We measured the protein level of GSDMD, the specific biomarker for pyroptosis. Our result showed that CLP induced an increase of GSDMD and that administration of MCC950 or Ac-YVAD-CMK reversed this increase, implying that the NLRP3/caspase-1 pathway mediates pyroptosis after CLP (Fig. 7a).

Caspase-1 regulates the cleavage and maturation of the downstream inflammatory cytokines IL-1β and IL-18. The CLP inflammatory process is associated with production of pro-inflammatory IL-1β and IL-18. To evaluate the effect of MCC950 and Ac-YVAD-CMK on hippocampal inflammation response, we measured the levels of IL-1β and IL-18 by Western blot and ELISA in SAE mice. Levels of IL-1β and IL-18 were significantly higher in the CLP+saline group compared to those in the sham+saline group, and administration of MCC950 or Ac-YVAD-CMK reversed this increase (Fig. 7a, b). Thus, inhibition of the NLRP3/caspase-1 pathway appears to alleviate inflammatory responses.

## DISCUSSION

In this study, we show that administration of either the NLRP3-inhibitor MCC950 or the caspase-1 inhibitor Ac-YVAD-CMK reduces mortality, reverses cognitive impairments, and rescues neuronal damages in mice that have undergone CLP. We further show the underlying mechanism of these effects; namely, the sepsis-induced formation of NLRP3 inflammasome leads to caspase-1 activation and triggers inflammatory cascades and pyroptosis. This supports the hypothesis that MCC950 and Ac-YVAD-CMK inhibit the NLRP3/caspase-1 pathway, alleviating pyroptosis and inflammation, and thus protecting mice from SAE.

Increasing evidence confirms that the brain can be affected during sepsis development, and that septic patients frequently suffer from cognitive impairments after discharge [3]. CLP appears to be a suitable clinical sepsis model and is an important tool by which to study cognitive impairment and its mechanism after sepsis [30]. Our results demonstrate that CLP decreases freezing time in the behavioral context test 24 h after training but not in the cue test, suggesting sepsis-induced hippocampus-dependent memory impairment in a mouse model of CLP, consistent with our previous investigation [14]. Our study also showed that sepsis does not induce anxiety-like behaviors in SAE mice, and this might be attributed to their full recovery without infection or motor alterations [19].

Neuroinflammation has been proposed as a possible pathogenic mechanism for SAE with long-term cognitive impairment [31, 32]. NLRP3 inflammasome is the most widely investigated inflammasome. It may activate caspase-1 leading to the processing and secretion of pro-inflammatory IL-1β and IL-18, which are implicated in several metabolic and inflammatory diseases [24, 33]. To date, NLRP3 inflammasome research has focused on the pathogenesis of a number of complex conditions, notably autoinflammation and autoimmune disease, that can be treated with the NLRP3-inhibitor MCC950 or the caspase-1 inhibitor Ac-YVAD-CMK [24–26]. In agreement with this, we found that increases of NLRP3, cleaved caspase-1, IL-1β, and IL-18 in the hippocampi of mice after CLP were reversed by MCC950 and that increases of cleaved caspase-1, IL-1β, and IL-18 were reversed by Ac-YVAD-CMK, indicating that CLP-induced activation of the NLRP3/caspase-1 pathway is inhibited by administration of MCC950 or Ac-YVAD-CMK. Most importantly, MCC950 or Ac-YVAD-CMK treatment prevented sepsis-induced neuronal damage and cognitive deficits in CLP mice, suggesting that the NLRP3/caspase-1 pathway is involved in the neurotoxicity and cognitive impairments observed in SAE. When NLRP3 is activated, sensor proteins oligomerize and recruit the adaptor protein ASC which then binds with caspase-1 to form the NLRP3 inflammasome [15]. Unexpectedly, CLP did not increase the expression of ASC in this model. It is possible that other proteins are involved in the formation of the NLRP3 inflammasome, such as NIMA-related kinases (NEK) [34] and protein kinase D (PKD) [35].

Pyroptosis is an inflammatory form of programmed cell death and thought to be involved in neuronal death in the hippocampus, an area of the brain important for learning and memory, leading to cognitive impairments [36]. Excessive pyroptosis causes sepsis and septic shock [27, 37]. Recent advances demonstrate that pyroptotic cell death is mediated by the NLRP3 inflammasome-caspase-1 pathway and that MCC950 and Ac-YVAD-CMK can inhibit the NLRP3 inflammasome response to prevent further pyroptosis [24–26]. GSDMD is the pivotal substrate of pyroptosis in sepsis [27, 37]. In this study, we show that the hippocampus of mice surviving CLP had higher GSDMD expressions than sham-operated groups and that the increase of GSDMD was reversed by administration of MCC950 or Ac-YVAD-CMK, providing further support for the role of pyroptosis in the pathogenesis of SAE. GSDMD lyses liposomes and forms pores on cell membranes; this, in turn, activates the NLRP3 inflammasome, driving caspase-1-dependent maturation of IL-1β, which is released from the cell upon membrane rupture [28, 38, 39]. Thus, we suggest that interactions among these proteins and cytokines tightly regulate pyroptosis and neuroinflammation responses. While little information about this exists, our data show that sepsis-triggered canonical inflammasome depends on the NLRP3/caspase-1 pathway for the maturation and secretion of IL-1β and on GSDMD for the induction of pyroptosis [40]. Notably, restoration of this pathway by administration of MCC950 or Ac-YVAD-CMK could reduce NLRP3-mediated overactivation of neuronal pyroptosis, downregulate the expression of mature IL-1β and IL-18, rescue neuronal damage, and reduce cognitive impairments in SAE mice.

Some limitations must be acknowledged in this study. First, no other specific biomarkers for neuronal pyroptosis in SAE were tested. Second, our experimental model only considers 14 days after CLP, based on a previous study [31], and long-term effects have not yet been considered. Finally, only five administration time points were used and no dose-response study for MCC950 or Ac-YVAD-CMK exists.

In summary, our data suggest the NLRP3/caspase-1 signaling pathway may play a vital role in neuronal pyroptosis and cognitive impairments in the development of SAE secondary to a sepsis. Restoration of the signaling pathway could reverse neurobehavioral abnormities. Inhibition of NLRP3 or caspase-1 provides one possible strategy to prevent and treat SAE.
